# The Cytokine and Bone Protein Expression by Ellagic Acid-Hydroxyapatite in Bone Remodelling Model

**DOI:** 10.1155/2022/6740853

**Published:** 2022-12-13

**Authors:** Dyah Noviana Primasari, Intan Nirwana, Hendrik Setia Budi, Agung Satria Wardhana, Annisa Fitria Sari, Novita Novita, Andika Putri Setyawan, Meircurius Dwi Condro Surboyo, Khairul Anuar Shariff

**Affiliations:** ^1^Department of Dental Materials, Faculty of Dental Medicine, Institute of Health Sciences Bhakti Wiyata, Kediri, Indonesia; ^2^Department of Dental Materials, Faculty of Dental Medicine, Universitas Airlangga, Surabaya, Indonesia; ^3^Department of Oral Biology, Faculty of Dental Medicine, Universitas Airlangga, Surabaya, Indonesia; ^4^Department of Dental Materials, Faculty of Dental Medicine, Universitas Lambung Mangkurat, Banjarmasin, Indonesia; ^5^Magister of Dental Science Program, Faculty of Dental Medicine, Universitas Airlangga, Surabaya, Indonesia; ^6^Dental Science Program, Faculty of Dental Medicine, Universitas Airlangga, Surabaya, Indonesia; ^7^Department of Oral Medicine, Faculty of Dental Medicine, Universitas Airlangga, Surabaya, Indonesia; ^8^Biomaterial Niche Area, School of Materials and Mineral Resource Engineering, Universiti Sains Malaysia, Pulau Pinang, Malaysia

## Abstract

**Objective:**

Ellagic acid, a phenolic compound with anti-inflammatory potential, can be used to accelerate the bone healing process and affect human health, while hydroxyapatite is the most commonly used bone graft material. Using a combination of the two materials results in reduced inflammation and increased osteogenesis. This study aimed to determine the effects of combining ellagic acid and hydroxyapatite in bone marker remodelling by analysing the expression of tumour necrosis factor-*α* (TNF-*α*), interleukin 10 (IL-10), bone morphogenetic 4 protein (BMP-4), and osteopontin (OPN).

**Methods:**

Thirty Wistar rats were used in the study. A defect was created in each animal's femur using a low-speed diamond bur. In the control group, the bone was then treated with polyethylene glycol (PEG). In one of the other groups, the bone was treated with hydroxyapatite, and in the other, with ellagic acid-hydroxyapatite. The femur was biopsied 7 days after the procedure and again 14 days after the procedure, and an indirect immunohistochemical (IHC) examination was performed for TNF-*α*, IL-10, BMP-4, and OPN expression.

**Results:**

The ellagic acid-hydroxyapatite decreased TNF-*α* expression in the bone tissue after 7 days and again after 14 days (*p* < 0.05). On the other hand, it increased IL-10, BMP-4, and OPN expression (*p* < 0.05) during the same time periods.

**Conclusion:**

Ellagic acid-hydroxyapatite plays a role in bone marker remodelling by decreasing the expression of TNF-*α* and increasing the expression of IL-10, BMP-4, and OPN. This hydroxyapatite combination can therefore be recommended for use as bone graft material.

## 1. Introduction

The alveolar bone is morphologically and functionally different from other bones. It is sensitive to mechanical stress and bone loss stimuli because it contains significantly different mineralisation rates than other bones in the body [1]. Alveolar bone defects occur easily and can necessitate several dental procedures, including tooth extractions. Tooth extractions commonly lead to further bone defects, especially in the anterior region of the maxilla where the cortical bone is thinner [2]. Alveolar bone retrieval procedures, such as odontectomies, also carry a risk of alveolar bone defects [3]. Excessive orthodontic pressure due to orthodontic treatment is another risk factor [4] as are pathological processes, such as periodontitis [5] and cleft lip and palate [6]. The aforementioned factors can result in bone defects that not only impede prosthetic reconstruction but that also cause aesthetic and masticatory problems [7]. As with other bone defects, alveolar defects can be repaired using a bone graft material.

Hydroxyapatite is commonly used as a bone graft material [8–10] because its chemical composition and crystal structure are similar to that of bone. In addition, hydroxyapatite has osteoconductive properties—it increases osteoblast activity while inhibiting excessive osteoclast activity [11]. The physical, chemical, and mechanical properties of hydroxyapatite, as well as the biocompatibility and bioactivity of the material, make it the most promising bone graft material in the field of modern dentistry [12, 13].

When prolonged inflammation occurs, the properties and effects of hydroxyapatite are no longer maximal. Prolonged inflammation leads to bone resorption activity due to osteoclast activity modulated by proinflammatory cytokines, such as TNF-*α* [14]. Therefore, adding an anti-inflammatory to the graft is necessary to suppress the inflammatory process. Ellagic acid may be used for this purpose. The dominant properties of ellagic acid are that it is an antioxidant and an anti-inflammatory [15–18]. For this reason, it is expected to reduce inflammation and stimulate bone remodelling [14, 19]. The study showed that ellagic acid-hydroxyapatite increases osteoblast activity, decreases osteoclast activity, and stimulates bone remodelling [20]. The decrease in osteoclast activity reflects the end stage of inflammation and the initial stage of bone deposition. During bone remodelling and deposition, anti-inflammatory cytokines and growth factors, such as interleukin 10 (IL-10), bone morphogenetic 4 protein (BMP-4), and osteopontin (OPN), are required to accelerate osteoblast maturation [21].

Due to the anti-inflammation properties of ellagic acid and the bone stimulation properties of hydroxyapatite, the aim of this research is to prove the efficacy of ellagic acid-hydroxyapatite in assisting and increasing bone marker remodelling. This will be achieved by analysing the expression of TNF-*α*, IL-10, BMP-4, and OPN. The results of the study will contribute to the research in the field of biomaterials and tissue regeneration, particularly as it relates to the orofacial region of the body.

## 2. Materials and Methods

### 2.1. Preparation of Hydroxyapatite and Ellagic Acid-Hydroxyapatite

For the purposes of the study, hydroxyapatite was converted into gel form by mixing hydroxyapatite (BATAN, Jakarta, Indonesia) and polyethylene glycol (PEG) (PEG, 202398, Sigma-Aldrich) (ratio 4 : 1 w/w). The ellagic acid-hydroxyapatite was formed by mixing ellagic acid (ellagic acid 90%, Xi'an Biof Bio-Technology, Shaanxi, China) and hydroxyapatite (ratio 93 : 7 w/w). This combination was then mixed with PEG (ratio 4 : 1 w/w) [20].

### 2.2. Animals

Thirty healthy male Wistar rats (*Rattus norvegicus*), each weighing 200–250 grams, were divided into three groups of five rats each.

### 2.3. Establishment of Bone Defects

The animals fasted for 12 hours before the procedure. They were placed under anaesthesia using ketamine hydrochloride (Ketalar, Warner–Lambert, Ireland) and xylazine (X1126, Sigma–Aldrich) (100 and 4 mg/kg body weight).

A 10 mm incision was made in each animal's lateral femur. The distance from the joint was 50 mm between the tibia and femur. The defect was created using a 0.84 mm round bur (801G/018, Meisinger, Germany), which rotated with a low-speed engine. The defect was 2 mm in diameter and depth. A saline solution was used for irrigation during the procedure.

After the bone defect was created, one of three materials was applied to the bone, depending on which group the animal was in. In one of the control groups, PEG was used; in another, hydroxyapatite was used; and in the experimental group, ellagic acid-hydroxyapatite was used.

After application, the tissue over the bone defect was sutured using nylon (Nylus nylon, nonabsorbable sutures, Lotus Surgicals, India), and the animals were given oral gentamicin (2–4 mg/kg body weight) every 24 hours. The animals received standard postsurgical care.

The femur of each animal was biopsied after 7 days and again after 14 days. An immunohistochemical (IHC) examination was also performed to analyse the defective bone tissue.

### 2.4. TNF-*α*, IL-10, BMP-4, and OPN Expression

An indirect IHC examination was carried out to analyse the bone tissue. The animals' macrophages were assessed for their TNF-*α* and IL-10 expression using TNF-*α* monoclonal antibodies (ab6671, rabbit polyclonal antibody, Abcam) and IL-10 monoclonal antibodies (ab34843, rabbit polyclonal antibody, Abcam). The BMP-4 and OPN expressions in the animals' osteoblast activity were assessed using BMP-4 monoclonal antibodies (ab39973, rabbit polyclonal antibody, Abcam) and OPN monoclonal antibodies (ab216402, rabbit polyclonal antibody, Abcam). Each expression was analysed using a light microscope with 400x magnification.

### 2.5. Data Analysis

The data were analysed using the Shapiro–Wilk test for data distribution and Levene's test for data homogeneity. The differences in expression between the control, hydroxyapatite, and ellagic acid-hydroxyapatite groups were analysed using the one-way analysis of variance (ANOVA) and Tukey's honest significant difference (HSD) tests. For the latter test, a test result of *p* < 0.05 was considered a significant difference.

## 3. Results

### 3.1. TNF-*α* Expression

**Figure 1:**
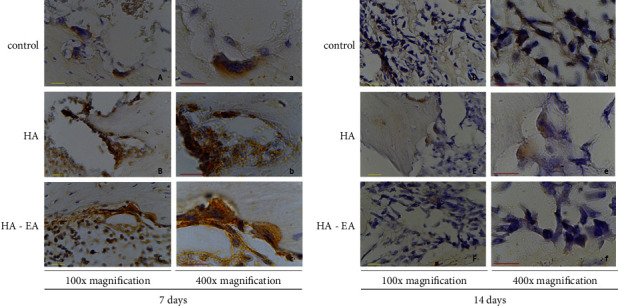
TNF-*α* expression in bone tissue 7 days after application (a, b, and c) and 14 days after application (d, e, and f). The TNF-*α* expression was expressed in macrophages as brown colour in figures.

**Figure 2:**
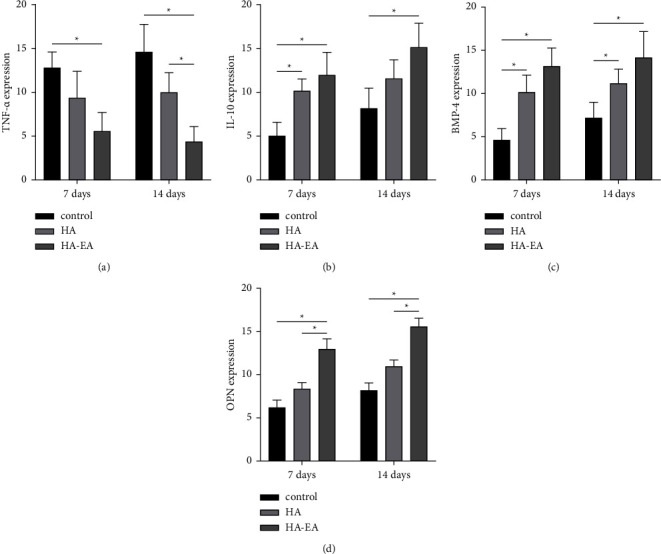
The mean of the bone marker expression in each group. The TNF-*α* (a), IL-10 (b), BMP-4 (c), and OPN (d) expressions. ^*∗*^ Indicate a value of significance using posthoc test as *p* < 0.05.

TNF-*α* expression was present in the bone tissue, as per Figure 1. The ellagic acid-hydroxyapatite group had lower TNF-*α* expression than the control group after seven days (*p* = 0.001). After 14 days, the ellagic acid-hydroxyapatite group had lower TNF-*α* expression than the hydroxyapatite and control groups (*p* = 0.001; *p* = 0.012) (See Figure 2(a)).

### 3.2. IL-10 Expression

**Figure 3:**
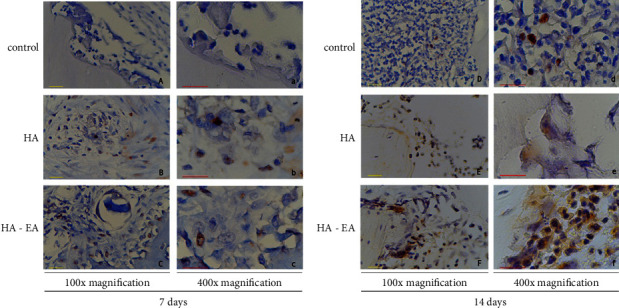
IL-10 expression in bone tissue 7 days after application (a, b, and c) and 14 days after application (d, e, and f). The IL-10 expression was expressed in macrophages as brown colour in figures.

IL-10 expression was present in the bone tissue, as per Figure 3. The ellagic acid-hydroxyapatite group had a higher expression than the control group after 7 days and again after 14 days (*p* = 0.001) (See Figure 2(b)).

### 3.3. BMP-4 Expression

**Figure 4:**
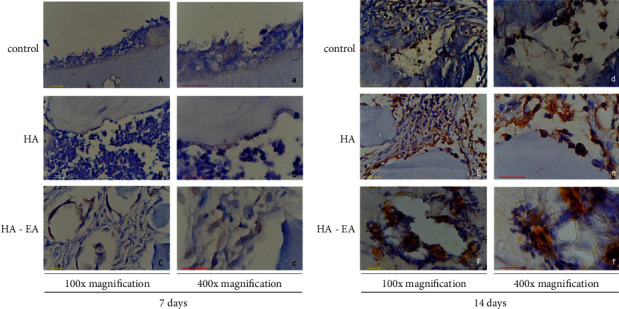
BMP-4 expression in bone tissue 7 days after application (a, b, and c) and 14 days after application (d, e, and f). The BMP-4 expression was expressed in osteoblast as brown colour in figures.

BMP-4 expression was present in the bone tissue, as per Figure 4. The ellagic acid-hydroxyapatite group had a higher expression than the control group after 7 days and again after 14 days (*p* = 0.001) (See Figure 2(d)).

### 3.4. OPN Expression

**Figure 5:**
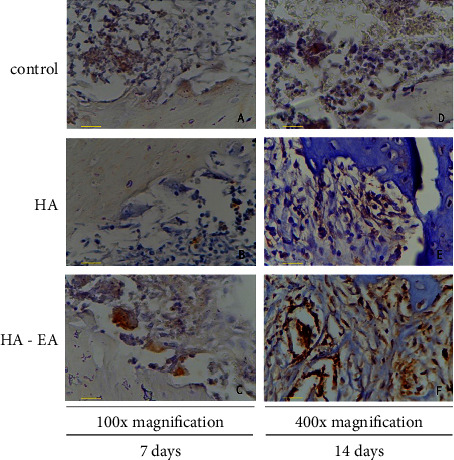
OPN expression in bone tissue 7 days after application (a, b, and c) and 14 days after application (d, e, and f). The OPN expression was expressed in osteoblast as brown colour in figures.

OPN expression was present in the bone tissue, as per Figure 5. The ellagic acid-hydroxyapatite group had the highest expression of all the groups after 7 days and again after 14 days (*p* < 0.05) (See Figure 2(c)).

## 4. Discussion

Inflammation is a normal part of the bone healing process. However, prolonged inflammation has numerous negative effects on the body, one being a prolonged healing process. Bone remodelling using only hydroxyapatite causes more intense inflammation and prolongs the healing process [22, 23] by producing TNF-*α* [24]. To decrease inflammation, hydroxyapatite should be combined with ellagic acid. This serves to control and suppress inflammation and predict the bone remodelling occurrence [25]. This condition proves that for the purposes of this research, ellagic acid-hydroxyapatite decreases TNF-*α* expression more effectively than hydroxyapatite. Uncontrolled TNF-*α* expression, due to prolonged inflammation, stimulates osteoclast differentiation, destroys the extracellular matrix, and results in bone resorption [26]. This indicates that ellagic acid reduces TNF-*α* expression by suppressing the activation of the nuclear factor kappa B (NF-*κ*B) pathway [27–30].

Ellagic acid in the NF-*κ*B pathway inhibits IKK activation to prevent degradation and reduce translocation to the nucleus. This process results in reduced proinflammatory cytokines, particularly TNF-*α* [31]. Inhibiting the production of proinflammatory cytokines increases anti-inflammatory cytokines and growth factors such as IL-10 and BMP-2 [31, 32].

IL-10 is a potent anti-inflammatory cytokine, which actively reduces and regulates other proinflammatory cytokines, such as TNF- *α* and interleukin 1*β* (IL-1*β*) [33, 34]. This study showed that IL-10 expression was higher in the bone tissue after 7 days and 14 days. The function of IL-10 is to regulate inflammation, which reduces proinflammatory cytokines, namely TNF-*α*. The higher proinflammatory cytokines (TNF-*α*) trigger osteoclast genesis and cause increased osteoclast differentiation, resulting in bone resorption. Increased IL-10 expression triggers osteoblast genesis by increasing osteoclast differentiation. This supports faster bone remodelling and accelerates all phases of bone healing. IL-10 appears to be an important regulator of bone homeostasis and inflammatory conditions [35, 36].

Other growth factors, such as the transforming growth factor, support bone remodelling, called BMP-4. BMP-4 regulates the migration and differentiation of mesenchymal stem cells during bone remodelling, induces osteogenesis, and rolls the remodelling of the bone matrix into mature bone [37]. In this study, BMP-4 expression increased as IL-10 expression increased. BMP-4 and IL-10 increased osteoblast activity's differentiation due to ellagic acid's anti-inflammatory properties [33]. The hydroxyapatite in the ellagic acid-hydroxyapatite combination also helps to increase bone remodelling. The hydroxyapatite releases calcium ions to support bone remineralisation [32].

The current research shows that the combination of ellagic acid-hydroxyapatite can reduce the main proinflammatory cytokine of bone inflammation, TNF-*α*, and increase bone growth factors, BMP-4 and OPN, through anti-inflammatory cytokine and IL-10. This mechanism is fundamental to initiate bone regeneration, to constitute the proportional ratio between osteoblast and osteoclast, as the primary cell in bone formation before cellular processes occur. The decreased TNF-*α*, in this research, also provides the finding that this cytokine may play a role in the decrease of osteoclast activity, as stated in the previous study [20]. Consequently, the decreased osteoclast activity will increase osteoblast activity, fostered by the anti-inflammatory properties of the ellagic acid and also lead to an increase in the bone protein proliferation factor, OPN. It is believed that OPN can induce mesenchymal stem cell (MSC) migration to defect sites and initiate differentiation into chondrocytes and osteoblasts [38]. This study also proved that ellagic acid-hydroxyapatite could increase OPN expression. The hydroxyapatite acts as a matrix to provide cell adhesion during remodelling [39].

Ellagic acid is several plants' polyphenolic compound, a secondary metabolite that is easy to obtain and commercially available as naturaceutical [40]. The source of ellagic acid is pomegranate (*Punica granatum* L.) and in the wood and bark of some tree species [41]. Of this availability, it will be easy to use and obtain and promising to use as a bone substitute with hydroxyapatite. The limitation of the study, it only analyzes one proinflammatory cytokine, namely TNF-*α*. Although other cytokines, such as IL-6 and IL-1*β*, also play a role. The TNF-*α* itself is representative to answer the purpose of this study because it is a marker of inflammation in general.

## 5. Conclusion

Current research shows that the combination of ellagic acid-hydroxyapatite combination is able to reduce the main proinflammatory cytokine of bone inflammation, TNF-*α* so that it can increase bone growth factors, BMP-4 and OPN, through IL-10. This mechanism is fundamental to initiate bone regeneration, before the constitution of osteoblast and osteoclast. This combination of materials can be safely recommended for bone grafting material to promote bone regeneration. Further study is needed, to implement this combination in humans, especially in bone fracture and orofacial bone defects, including alveolar bone defects, cleft lip, and palate. Regarding safety, some studies have shown such low toxicity that the chances of use are even greater.

## Data Availability

The data will be available upon personal request to address to the corresponding author Intan Nirwana (intan-n@fkg.unair.ac.id).

## Additional Points

Ellagic acid-hydroxyapatite plays a role in bone marker remodelling by decreasing the expression of TNF-*α*. Ellagic acid-hydroxyapatite also plays a role in bone marker remodelling by increasing the expression of IL-10, BMP-4, and OPN.
